# Neuropathic pain in peroneal nerve entrapment at the fibular head

**DOI:** 10.1055/s-0042-1758644

**Published:** 2022-12-28

**Authors:** İlker Öztürk, Halit Fidanci, Zülfikar Arlier

**Affiliations:** 1 Adana City Training and Research Hospital, Department of Neurology, Adana, Turkey.; 2 Adana City Training and Research Hospital, Department of Neurology, Division of Clinical Neurophysiology, Adana, Turkey.

**Keywords:** Electrodiagnosis, Neural Conduction, Neuralgia, Peroneal Nerve, Eletrodiagnóstico, Condução Nervosa, Neuralgia, Nervo Fibular

## Abstract

**Background** Peroneal neuropathy at the fibular head (PNFH) is a mononeuropathy that typically presents with drop foot and sensory abnormalities over the skin area innervated by the peroneal nerve.

**Objective** The aim of the present study was to evaluate neuropathic pain in patients with PNFH.

**Methods** Patients with clinical and electrodiagnostic features consistent with PNFH associated with weight loss, leg postures, or prolonged sleep were included in the present retrospective cohort study. Nerve conduction studies were performed in the bilateral lower extremities of all patients. The Leeds assessment of neuropathic symptoms and signs scale (LANSS) was applied to all patients.

**Results** Thirty-two PNFH patients (78% males) were included in the study. The LANSS score in the majority of patients was lower than 12. There was 1 patient with a LANSS score of 12. The electrodiagnostic features of 16 patients were compatible with axonal degeneration. The mean LANSS scores of PNFH patients with and without axonal degeneration were 4.3 ± 3.7 and 5.2 ± 2.9, respectively ( *p* = 0.255).

**Conclusion** The present study showed that neuropathic pain is a rare symptom in patients with PNFH associated with weight loss, leg postures, or prolonged sleep.

## Resumo

**Antecedentes** A neuropatia fibular na cabeça da fíbula (PNFH) é uma mononeuropatia que normalmente se apresenta com pé caído e anormalidades sensoriais sobre a área da pele inervada pelo nervo fibular.

**Objetivo** O objetivo do presente estudo foi avaliar a dor neuropática em pacientes com PNFH.

**Métodos** Pacientes com características clínicas e eletrodiagnósticas consistentes com PNFH associada a perda de peso, postura das pernas ou sono prolongado foram incluídos neste estudo de coorte retrospectivo. Estudos de condução nervosa foram realizados nas extremidades inferiores bilaterais de todos os pacientes. A escala de avaliação de sintomas e sinais neuropáticos de Leeds (LANSS) foi aplicada a todos os pacientes.

**Resultados** Trinta e dois pacientes com PNFH (78%) foram incluídos no estudo. A pontuação LANSS em outros pacientes foi menor que 12. Houve 1 paciente com pontuação LANSS de 12. As características eletrodiagnósticas de 16 pacientes foram compatíveis com degeneração axonal. Os escores médios do LANSS de pacientes com PNFH com e sem degeneração axonal foram 4,3 ± 3,7 e 5,2 ± 2,9, respectivamente (p = 0,255).

**Conclusão** O presente estudo mostrou que a dor neuropática é um sintoma raro em pacientes com PNFH associada à perda de peso, postura das pernas ou sono prolongado.

## INTRODUCTION

Peroneal neuropathy at the fibular head (PNFH) is the most common entrapment mononeuropathy of the lower extremities, characterized by drop foot and sensory abnormalities over the skin area supplied by the peroneal nerve. 1 2 3 Trauma, or disorders such as diabetes mellitus can cause PNFH. 1 2 3 4 In addition, weight loss and prolonged repetitive leg postures, such as crossing legs or squatting, are known to be associated with PNFH. 1 3 4 5 6 7

Peroneal neuropathy at the fibular head can be diagnosed with electrodiagnostic and clinical findings. 1 2 3 4 Nerve conduction studies for PNFH typically find motor conduction blocks and/or the slowing of the motor nerve conduction velocity (NCV) of the peroneal nerve across the below fibular head-popliteal fossa (BFH-PF) segment. 1 2 3 4 Clinical signs of PNFH often include weakness in foot dorsiflexion and/or ankle eversion, as well as sensory abnormalities over the skin area innervated by the peroneal nerve. 4 While pain can be an important symptom in some mononeuropathies (such as carpal tunnel syndrome and sciatic nerve injury due to intramuscular injection), 8 9 it appears to be less common in others (such as elbow ulnar neuropathy). 10 The aim of the present study was to evaluate neuropathic pain in patients with PNFH and thus to obtain information about the pathophysiology of PNFH.

## METHODS

### Study design and subjects

Individuals who applied to our clinical neurophysiology laboratory between July 2018 and October 2020 and whose both clinical and electrodiagnostic findings were compatible with PNFH due to external compression or weight loss were included in the present retrospective cohort study. Since PNFH due to weight loss or external compression is common, patients with these conditions were deemed appropriate to be included in the present study. 1 2 3 Ethics committee approval was received from the Adana City Training and Research Hospital Clinical Research Ethics Committee (number 68/1108). If either or both symptoms of weakness in the muscles innervated by the peroneal nerve or sensory abnormality over the skin area supplied by the peroneal nerve were detected during the neurological examination, the clinical findings of the patient were considered to be compatible with PNFH. Patients with any of the following conditions or diseases were excluded from the study: polyneuropathy (or a disease that could cause polyneuropathy, such as diabetes mellitus); PNFH associated with masses; neurodegenerative disease; lumbosacral radiculopathy/plexopathy; sciatic neuropathy; or a family history of neurodegenerative or hereditary polyneuropathy. Knee/leg magnetic resonance imaging (MRI) and/or knee/leg ultrasonography were performed in all patients. In addition, lumbosacral MRI findings of all patients were analyzed to exclude lumbosacral radiculopathy. Muscle strength was scored using the Medical Research Council scale (MRC). 11 The Turkish version of the Leeds assessment of neuropathic symptoms and signs (LANSS) was used to assess neuropathic pain in the PNFH patients. 12 If the LANSS score was ≥ 12, it was decided that the patient had neuropathic pain. 12 The LANSS, electrodiagnostic tests, and neurological examination were performed at the same time.

### Electrodiagnostic tests

The nerve conduction study and the needle electromyography (EMG) were performed with a Cadwell Sierra Summit EMG unit (Cadwell Laboratories, Kennewick, WA, USA). Electrodiagnostic tests were applied to patients when the temperature of their extremities was ≥ 32°C; cold extremities were heated. Surface electrodes were used for recording and stimulation, which was performed supramaximally. The duration of stimulation for the nerve conduction studies was 0.1 ms. The low-high band filters were set at 20 Hz to 2 kHz and at 20 Hz to10 kHz for sensory nerve conduction and motor nerve conduction studies, respectively. Sweep speed and sensitivity for sensory nerve conduction studies were 1 ms/division and 10 µV/division, respectively. Sweep speed and sensitivity for motor nerve conduction studies were set at 5 ms/division and 2 mV/division, respectively. Compound muscle action potential (CMAP) and compound nerve action potential (CNAP) amplitudes were measured from peak to peak. Superficial peroneal sensory NCV was calculated using onset latency.

Peroneal, posterior tibial, superficial peroneal, and sural nerve conduction studies of both lower extremities, as well as median and ulnar nerve conduction studies of one upper extremity, were applied to all patients. The reference values of our clinical neurophysiology laboratory were used for the nerve conduction study. 13 14 Peroneal nerve compound muscle action potential (CMAP) was obtained by recording from both the extensor digitorum brevis (EDB) and tibialis anterior (TA) muscles. Posterior tibial nerve CMAP was recorded from the abductor hallucis muscle. For peroneal and posterior tibial nerve stimulation at the ankle, the distance between the recording electrode and the stimulation point was 8 cm and 10 cm, respectively. The peroneal nerve stimulation points were the ankle, below the fibular head, and the popliteal fossa. The distance between the recording electrode and the stimulation point was 12 to 14 cm for the superficial peroneal sensory nerve conduction study. The lower or upper limits of reference values for peroneal motor nerve conduction and superficial peroneal sensory nerve conduction studies were as follows 13 : peroneal nerve CMAP terminal latency < 5.2 m/s (EDB); peroneal nerve terminal CMAP amplitude > 3.7 mV (EDB) /> 3.9 mV (TA); peroneal motor NCV across ankle - below fibular head segment > 43.9 m/s (EDB); peroneal motor NCV across the BFH-PF segment > 40.1 m/s (EDB) /> 41.0 m/s (TA); superficial peroneal CNAP amplitude / velocity > 5.3 µV /> 37.0 m/s. In addition, peroneal motor NCV (EDB) was considered abnormal if peroneal motor NCV (EDB) across the BFH-PF segment was > 6 m/s slower than peroneal motor NCV (EDB) across ankle/below fibular head segment or if peroneal motor NCV (EDB) across the BFH-PF segment decreased by > 12% compared with the peroneal motor NCV (EDB) of the across-ankle/below fibular head segment. 15 If the CMAP amplitude obtained by peroneal motor nerve stimulation at the popliteal fossa decreased by > 25% compared with the CMAP amplitude obtained by peroneal motor nerve stimulation below the fibular head, it was considered abnormal. 15 If the peroneal nerve CMAP or superficial peroneal CNAP amplitude were reduced, axonal degeneration was considered. 1 2 The duration of CMAP was taken into account when deciding whether there was axonal degeneration or not. The patients were divided into two groups according to their electrodiagnostic findings as those with and without axonal degeneration.

The low-high band filter for the needle EMG was set at 10Hz to 10kHz. Sweep speed and sensitivity for analysis of positive sharp wave (PSW) and fibrillation potentials were set at 10 ms/division and 100 µV/division, respectively. Sweep speed and sensitivity for motor unit action potential (MUAP) analysis were 10 ms/division and 500-1000 µV/division, respectively. For the needle EMG, a concentric needle electrode (length = 50mm, diameter = 0.46mm, Bionen Medical Devices, Florence, Italy) was used. The needle EMG was performed visually. The presence of active denervation was carefully analyzed. Active denervation severity was scored as follows: no active denervation = 0; a PSW or fibrillation potential in at least 2 areas = 1; moderate numbers of PSW or fibrillation potentials in 3 or 4 areas = 2; many PSW or fibrillation potentials in all areas = 3; and PSW or fibrillation potentials that filled the entire screen in all areas = 4. Ten to 20 MUAPs were analyzed in each muscle. Motor unit action potential analysis was performed during mild muscle contraction. Neurogenic MUAP was considered if the MUAP amplitude was > 4 mV and the MUAP duration was > 15 ms. The needle EMG was applied to the following muscles in all patients: tibialis anterior, peroneus longus, medial gastrocnemius, short head of biceps femoris, vastus lateralis, and L3, L4, L5, and S1 paraspinal muscles. Patients whose nerve conduction study and needle EMG findings were compatible with polyneuropathy, or lumbosacral plexopathy/radiculopathy were not included in the study.

### Statistical analysis

The Shapiro-Wilk test was used to determine the distribution of the data. The Pearson chi-squared and the Fisher exact tests were used to analyze categorical variables. The Mann-Whitney U test was used in group comparisons. Categorical variables were summarized as percentage and frequency. The mean ± standard deviation (SD) and minimum-maximum of the numeric data were calculated for descriptive statistics. The Spearman test was used for correlation. If the *p-value* was < 0.05, it was considered statistically significant. IBM SPSS Statistics for Windows version 22.0 (IBM Corp., Armonk, NY, USA) was used to perform the statistical analysis.

## RESULTS

**Figure 1.**
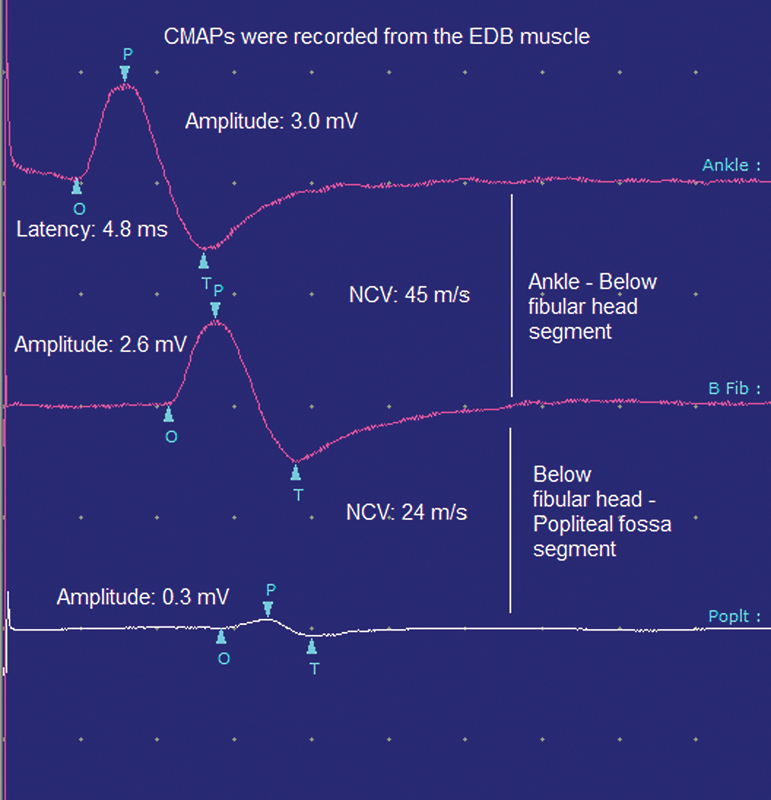
Motor conduction block of the right peroneal nerve across-below the fibular head-popliteal fossa segment in a PNFH patient with a history of weight loss. EDB, extensor digitorum brevis; CMAP, compound muscle action potential; NCV, nerve conduction velocity; PNFH, peroneal neuropathy at the fibular head (Sweep speed: 5 ms/division, sensitivity: 2 mV/division).

Thirty-two PNFH patients (78% males) were included in the study. The mean age of the patients was 31.6 ± 15.2 (range 15–82) years old. The mean height, weight, and body mass index (BMI) of the patients were 176.6 ± 7.9 (range 160–194) cm, 68.9 ± 12.4 (range 40–85) kg, and 21.9 ± 3.2 (range 15.2–27.8) kg/m ^2^ , respectively. Five patients (16%) had a BMI < 18.5 kg/m ^2^ and none of the patients had a BMI > 30 kg/m ^2^ . The clinical characteristics of the patients are shown in Table 1 . The mean amount of weight loss per month for the 11 patients who lost weight was 5.2 ± 2.5 kg (2.5–10.0 kg). The mean MRC scores of foot dorsiflexion and ankle eversion were 2.7 ± 1.4 and 3.0 ± 1.7, respectively. The electrodiagnostic findings of the patients are shown in Table 2 . Table 3 shows the electrodiagnostic abnormalities in the patients. An example of motor conduction block across the BFH-PF segment of the peroneal nerve of a PNFH patient is shown in Figure 1 . The interval between the onset of symptoms and the application of electrodiagnostic tests and LANSS to the patients was 35.4 ± 13.4 (range 21–60) days. This interval was ≤ 30 days in 22 patients.

**Table 1:** Clinical features of the patients

Clinical feature		Number of patients (%)
Male		25 (78.1)
Right-sided PNFH		20 (62.5)
Medical condition	Crossing legs	14 (43.8)
	Squatting	7 (21.9)
	Prolonged sleep	5 (15.6)
	Weight loss	5 (15.6)
	Both crossing legs and weight loss	6 (18.8)
	Prolonged sleep	5 (15.6)
Neurological examination - weakness	Dorsiflexion of foot	31 (96.9)
	Eversion of foot	26 (81.3)
	None	1 (3.1)
Neurological examination - sensory abnormality	Dorsum of foot	10 (31.3)
	Lateral of leg	1 (3.1)
	Both foot dorsum and leg lateral	15 (46.9)
	None	6 (18.8)

Abbreviation: PNFH, peroneal neuropathy at the fibular head.

**Table 2:** Electrodiagnostic findings of the patients

Electrodiagnostic parameter	Mean ± SD (min-max) (number)
Terminal CMAP amplitude (mV) EDB / TA	6.2 ± 2.8 (0–11.9) ( *n* = 32) / 7.5 ± 3.3 (2.1–14.5) ( *n* = 32)
Motor NCV across BFH-PF segment (m/s) EDB† /TA‡	36.7 ± 7.5 (22 *–* 53) ( *n =* 31) / 39.2 ± 11.7 (20 *–* 65) ( *n =* 31)
CMAP amplitude reduction in percentage across BFH-PF segment (%) EDB† / TA	58.7 ± 32.1 (0 *–* 98.1) ( *n =* 31) / 53.1 ± 26.6 (0 *–* 100) ( *n =* 32)
Motor NCV difference between ankle-BFH and BFH-PF segments EDB (m/s)†	11.1 ± 6.9 (2 *–* 27) ( *n =* 31)
Reduction of motor NCV across the BFH-PF segment compared with that across the ankle-below fibular head segment (%)†	23.1 ± 13.8 (4.2 *–* 51.1) ( *n =* 31)
Superficial peroneal CNAP amplitude (µV)	12.6 ± 8.4 (0 *–* 27.7) ( *n =* 32)
Superficial peroneal sensory NCV (m/s) §	48.4 ± 5.8 (37.0 *–* 57.5) ( *n =* 28)
The severity of active denervation – TA	2.5 ± 0.8 (1 *–* 4)
The severity of active denervation – PL	1.3 ± 0.5 (0 *–* 3)

Abbreviations: BFH-PF, below fibular head-popliteal fossa; CMAP, compound muscle action potential; CNAP, compound nerve action potential; EDB, extensor digitorum brevis; NCV, nerve conduction velocity; PL, peroneus longus; TA, tibialis anterior; SD, standard deviation.

Notes: †, Since peroneal nerve CMAP (EDB) could not be obtained in one patient, this patient was not included.

‡, Since peroneal nerve CMAP (TA) could not be obtained in one patient by peroneal nerve stimulation at the popliteal fossa, this patient was not included.

§, Since superficial peroneal nerve CNAP could not be obtained in three patients, these patients were not included.

**Table 3:** Electrodiagnostic abnormalities in patients

Electrodiagnostic parameters	Number of patients (%)
CMAP amplitude (mV) < 3.7 mV (EDB)	13 (40.6)
CMAP amplitude (mV) < 3.9 mV (TA)	9 (28.1)
Motor NCV across BFH-PF segment < 40.1 m/s (EDB)†	20 (64.5)
Motor NCV across BFH-PF segment < 41.0 m/s (TA)‡	19 (61.3)
Motor NCV across BFH-PF segment < 40.1 m/s (EDB) or < 41 m/s (TA)	32 (75.0)
CMAP amplitude reduction in percentage across BFH-PF segment > 25% (EDB)†	24 (77.4)
CMAP amplitude reduction in percentage across BFH-PF segment > 25% (TA)	29 (90.6)
CMAP amplitude reduction in percentage across BFH-PF segment > 25% (EDB or TA)	31 (96.9)
Motor NCV difference between ankle-below fibular head and BFH-PF segments > 6 m/s (EDB)†	20 (64.5)
Reduction of motor NCV across the BFH-PF segment compared with that across the ankle-BFH segment > 12% (EDB)†	22 (71.0)
Superficial peroneal CNAP amplitude < 5.3 µV	8 (25)
Superficial peroneal sensory NCV < 37.0 m/s§	1 (3.6)
PSW or fibrillation potentials – TA	32 (100)
PSW or fibrillation potentials – PL	23 (71.9)
Neurogenic MUAP – TA	1 (3.1)

Abbreviations: BFH-PF, below fibular head-popliteal fossa; CMAP, compound muscle action potential; CNAP, compound nerve action potential; EDB, extensor digitorum brevis; MUAP, motor unit action potential; NCV, nerve conduction velocity; PL, peroneus longus; TA, tibialis anterior; SD, standard deviation.

Notes: † Since peroneal nerve CMAP (EDB) could not be obtained in one patient, this patient was not included.

‡ Since peroneal nerve CMAP (TA) could not be obtained in one patient by peroneal nerve stimulation at the popliteal fossa, this patient was not included.

§ Since superficial peroneal nerve CNAP could not be obtained in three patients, these patients were not included.

**Figure 2.**
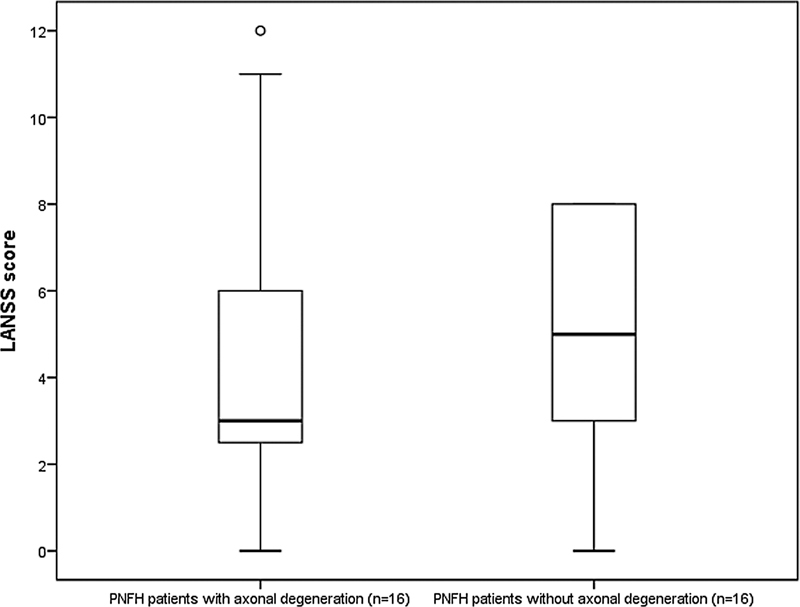
Comparison of LANSS scores between PNFH patients with and without axonal damage. The mean LANSS scores of PNFH patients with and without axonal degeneration were 4.3 ± 3.7 (min-max 0-12) and 5.2 ± 2.9 (min-max 0-8), respectively ( *p* = 0.255, Mann Whitney U test). LANSS, the Leeds assessment of neuropathic symptoms and signs; PNFH, peroneal neuropathy at the fibular head.

The mean LANSS score was 4.7 ± 3.3 (range 0–12) Only 1 patient had a LANSS score of 12, while the other patients had a LANSS score < 12. The number of PNFH patients with axonal degeneration was 16. The comparison of LANSS scores of patients with and without axonal injury is shown in Figure 2 . There was no significant difference in LANSS scores between patients with and without superficial peroneal CNAP amplitude abnormality ( *p* = 0.756). Similarly, LANSS scores of the patients with and without peroneal nerve CMAP amplitude abnormality recorded from TA/EDB muscles were not significantly different ( *p* = 0.399/ *p* = 357). Table 4 shows the correlation analysis between LANSS scores and electrodiagnostic parameters. The patient with a LANSS score of 12 was female and 18 years old. This patient had a history of repetitively crossing legs. Neurological examination, LANSS and electrodiagnostic tests were performed 30 days after the onset of her complaints. Four patients had allodynia. Superficial peroneal nerve SNAP amplitude and NCV were normal in these patients. In two of these four patients, reduced peroneal nerve CMAP amplitude (recorded from EDB and TA muscles) was found.

**Table 4:** Correlation between MRC scores / electrodiagnostic parameters and LANSS scores

Clinical and electrodiagnostic parameter	LANSS score	
	P	R
Age	0.576	0.103
Duration	0.997	0.001
MRC score of DF	0.330	-0.178
MRC score of eversion	0.305	−0.187
Peroneal nerve CMAP amplitude (EDB)	0.203	0.231
Peroneal nerve CMAP amplitude (TA)	0.846	0.036
Motor NCV across BFH-PF segment (EDB)†	0.090	−0.310
Motor NCV across BFH-PF segment (TA)‡	0.991	0.002
CMAP amplitude reduction in percentage across BFH-PF segment (EDB)†	0.465	0.136
CMAP amplitude reduction in percentage across BFH-PF segment (TA)	0.991	0.002
Superficial peroneal CNAP amplitude	0.621	−0.091
Superficial peroneal sensory NCV§	0.663	−0.086
The severity of active denervation – TA	0.888	0.026
The severity of active denervation – PL	0.398	0.155

Abbreviations: BFH-PF, below fibular head-popliteal fossa; CMAP, compound muscle action potential; CNAP, compound nerve action potential; EDB, extensor digitorum brevis; LANSS, the Leeds assessment of neuropathic symptoms and signs; MRC, Medical Research Council scale; NCV, nerve conduction velocity; PL, peroneus longus; TA, tibialis anterior.

Notes: † Since peroneal nerve CMAP (EDB) could not be obtained in one patient, this patient was not included.

‡ Since peroneal nerve CMAP (TA) could not be obtained in one patient by peroneal nerve stimulation at the popliteal fossa, this patient was not included.

§ Since superficial peroneal nerve CNAP could not be obtained in three patients, these patients were not included. Spearman's correlation test was used.

## DISCUSSION

The two most typical clinical indicators of PNFH are weakness of the peroneal innervated muscles and sensory abnormalities over the skin area innervated by the peroneal nerve. 1 2 3 4 In the present study, ∼ 95% of the patients demonstrated weakness in the TA muscle innervated by the deep peroneal nerve, while ∼ 80% showed weakness in the peroneus longus muscle innervated by the superficial peroneal nerve. This finding may indicate that the peroneal nerve fascicles innervating the muscles are affected separately in PNFH. 3 16

In the present study, superficial peroneal CNAP abnormality was present in six patients. It is known that injuries to sensory nerves can cause neuropathic pain. 17 18 The fact that the superficial peroneal nerve CNAP amplitude abnormality was found in a small number of patients may mean that this nerve has little or no axonal degeneration. The frequent presence of the conduction block and its temporary nature may cause the sensory fibers to be less likely to be affected. Sprouting, which is accepted as one of the neuropathic pain mechanisms as a result of peripheral nerve damage, may therefore not occur in PNFH. 18 19 This condition and the fact that sensory abnormalities were not evident in PNFH patients when compared with motor deficits may also support that neuropathic pain is a rare finding in PNFH. In addition to sprouting, conditions such as central mechanisms, peripheral nervous system sensitization, increased sodium channels are involved in the pathophysiology of neuropathic pain. 18 19 The disappearance of the conduction block in PNFH in a short time and the lack of time for these conditions to occur may explain the lack of neuropathic pain in PNFH. Leeds assessment of neuropathic symptoms and signs scores were not 12 or higher except for in 1 patient. The presence of allodynia in only four patients may also indicate that neuropathic pain is rarely found in PNFH.

Electrodiagnostic tests play an important role in the diagnosis of PNFH. The most common nerve conduction study abnormality found in PNFH in the present study was peroneal nerve motor conduction block at the BFH-PF segment. 1 20 Recording CMAP from both TA and EDB muscles may increase the sensitivity of PNFH diagnosis. This finding and the more pronounced needle EMG abnormality in the TA muscle compared with the peroneus longus muscle may be due to the topographic distribution of the peroneal nerve fascicles mentioned earlier. 3 16

Although there are controversial results, it is more likely that there is no relationship between electrodiagnostic findings and neuropathic pain. 10 21 22 Similar to some previous studies conducted on entrapment neuropathies, we did not find a relationship between neuropathic pain and electrodiagnostic findings in PNFH. 10 21 22 These findings may also support that neuropathic pain is rare in PNFH. But this may be related to the time of performing electrodiagnostic tests and the ability to test the large nerve fibers of routine nerve conduction studies. 21 23 In addition, nerve conduction study findings may vary according to the time of the electrodiagnostic test. Therefore, the improvement of parameters such as motor conduction block or nerve conduction velocity in a short time may have caused less or no neuropathic pain in PNFH.

It is known that PNFH is often associated with external pressure or weight loss. 1 2 3 Therefore, patients with PNFH associated with weight loss, prolonged repetitive leg posture, or after prolonged sleep were included in the present study. The fact that neuropathic pain was found at a low rate in PNFH in the present study may be related with the etiology of PNFH. Pain may be an important symptom in peroneal neuropathy caused by heavy exertion or trauma in athletes and in peroneal neuropathy due to ganglion cysts. 24 25 We think that a study involving patients with peroneal neuropathy due to different etiologies, such as patients with peroneal neuropathy due to trauma, would be interesting and relevant. Pain may be prominent in different mononeuropathies due to different etiologies. Pain is an important symptom in sciatic nerve injury due to intramuscular injection and carpal tunnel syndrome. 8 9 13

The present study had some limitations. First, the symptom durations of the patients were different from each other. This may have affected LANSS scores and electrodiagnostic findings. Even with these differences, the interval between the onset and evaluation of symptoms was ≤ 30 days in most patients. Second, A delta and C fibers associated with neuropathic pain cannot be evaluated with routine nerve conduction studies. This may explain why there was no correlation between LANSS scores and electrodiagnostic findings in the present study. Third, patients with PNFH due to a certain etiology as we mentioned earlier were included.

The present study showed that neuropathic pain was a rare symptom in PNFH associated with weight loss, prolonged repetitive leg postures, or prolonged sleep. It was also revealed that there was no relationship between LANSS scores and electrodiagnostic parameters in PNFH. This may mean that demyelination is predominant in PNFH due to conditions such as leg posture. Studies on neuropathic pain in PNFH due to different etiologies may provide an opportunity to understand the pathophysiology of neuropathic pain.
